# Mixed cerebrovascular disease in an elderly patient with mixed vascular risk factors: a case report

**DOI:** 10.1186/s12883-019-1248-z

**Published:** 2019-02-12

**Authors:** Dian He, YunLi Yu, Shan Wu, ShuFen Tian, Hui Yu, Shu Xu, Lan Chu

**Affiliations:** 1 Department of Neurology, Affiliated Hospital of Guizhou Medical University, Guiyang, Guizhou Province China; 2 Department of Radiology, Affiliated Hospital of Guizhou Medical University, Guiyang, Guizhou Province China; 3 Department of Pathology, Affiliated Hospital of Guizhou Medical University, Guiyang, Guizhou Province China

## Abstract

**Background:**

Mixed cerebrovascular disease is a diagnostic entity that presents with hemorrhagic and ischemic stroke clinically and/or subclinically. Here, we report a patient with mixed vascular risk factors, who presented with multiple intracerebral hemorrhages and a simultaneously occurring cerebral infarction with hemorrhagic transformation.

**Case presentation:**

A 63-year-old male with no history of trauma or prior neurological disease presented with a sudden onset of weakness in his right limbs, followed by an episode of focal seizure without impaired awareness. The patient had a 4-year history of deep vein thrombosis in the lower limbs, and a 2-year history of Raynaud’s phenomenon in the hands. He also had a family history of hypertension and thrombophilia. Head computed tomography plain scans showed two high densities in the bilateral parietal lobes and one mixed density in the left frontal lobe. The patient was diagnosed with mixed cerebrovascular disease. In this report, we make a systematic clinical reasoning regarding the etiological diagnosis, and discuss the possible pathogenic mechanisms leading to mixed cerebrovascular disease. We exclude coagulopathy, endocarditis, atrial fibrillation, patent foramen ovale, brain tumor, cerebral venous thrombosis, cerebral vascular malformation, cerebral amyloid angiopathy and vasculitis as causative factors. We identify hypertension, hereditary protein S deficiency, hypercholesteremia and hyperhomocysteinemia as contributing etiologies in this case.

**Conclusion:**

This case presents complex underlying mechanisms of mixed cerebrovascular disease, in which hypertension and hyperhomocysteinemia are considered to play a central role.

## Background

Stroke is routinely classified into two main groups: ischemic and hemorrhagic. In many cases, however, ischemic and hemorrhagic cerebrovascular disease coexist, in which case it is termed as “mixed cerebrovascular disease (MCVD)”. MCVD represents a diagnostic entity that presents with hemorrhagic and ischemic stroke clinically and/or subclinically, including clinical ischemic stroke, subclinical cerebral infarct, cerebral white matter disease, intracerebral hemorrhage (ICH), and cerebral microbleeds (CMBs) [1]. MCVD can have complex underlying mechanisms, making successful treatment challenging [2]. Herein, we report a patient with multiple vascular risk factors, who presented with multiple ICHs and a cerebral infarction with hemorrhagic transformation that occurred simultaneously. We make a systematic clinical reasoning for the etiological diagnosis of MCVD for this case, and discuss the possible pathogenic mechanisms.

## Case presentation

A 63-year-old male with no history of trauma or prior neurological disease presented with 3 days of intermittent dizziness and vomiting. On the second day, the patient was examined by a head computed tomography (CT) scan, and no abnormal changes were found. Five days later, the patient had a sudden onset of weakness in his right limbs, followed by an episode of focal seizure without impaired awareness. The patient had symptomatic deep venous thrombosis (DVT) in the left lower limb 4 years ago, which was treated with warfarin for 3 months. No secondary prophylaxis was subsequently applied, and as a result he experienced multiple recurrences of lower limb DVT. The patient also had a 5-year history of hypertension without antihypertensive therapy. He also had a 2-year history of Raynaud’s phenomenon in his hands, as well as a 30-year history of smoking (10 cigarettes per day) and alcohol intake (50 g per day). The patient’s parents had died of ICH. Additionally, the patient’s three sisters were diagnosed with hypertension, and one brother had a history of occlusion of the distal artery in the right leg at the age of 55 years. The patient’s son had their first symptomatic lower limb DVT at the age of 25 years, for which he underwent inferior vena caval filter placement.

**Fig. 1:**
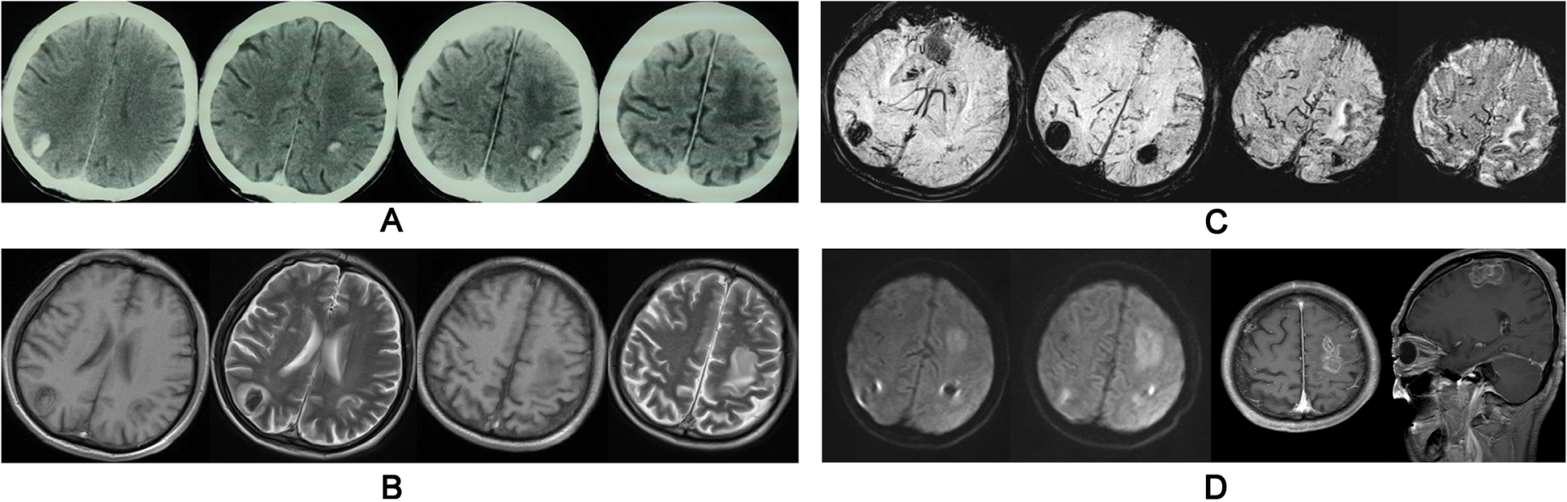
Lesions displayed on the head CT and MRI.Multiple hemorrhages located in the bilateral parietal lobes were revealed, as well as an infarction with hemorrhagic transformation in the left frontal lobe on the head plain CT (**a**), T1WI/T2WI (**b**), SWI (**c**), DWI (**d**, first two images), and gadolinium-enhanced MRI (**d**, last two images)

Upon examination after admission, the patient had a blood pressure of 164/92 mmHg. Skin color, temperature, and peripheral pulses were normal. No varicose veins or swelling of the limbs were found, and the lung and heart examinations were normal. The patient was fully alert and oriented, with no signs of cognitive impairment, thus neurocognitive tests were not performed. Results of the cranial nerve and sensory examinations were normal. Motor examination revealed spastic tone and moderate pyramidal weakness in the right arm and leg (4/5), with a total NIHSS score of 2. Repeated head CT plain scans (Fig. 1a) and brain magnetic resonance imaging (MRI) plain scans (Fig. 1b) showed acute hematomas in the bilateral parietal lobes, and a cerebral infarction with hemorrhagic transformation in the left frontal lobe. Except for the hemorrhages visible on the head CT, brain susceptibility-weighted imaging (SWI) showed neither venous malformations nor CMBs (Fig. 1c). Brain diffusion-weighted imaging (DWI; Fig. 1d, first two images) and contrast-enhanced MRI (Fig. 1d, last two images) confirmed the cerebral infarction with hemorrhagic transformation in the left frontal lobe. The degree of white matter hyperintensity (WMH) on fluid attenuated inversion recovery (FLAIR) imaging was moderate (Grade II on the Fazekas scale). The results of brain magnetic resonance angiography (MRA) and magnetic resonance venography (MRV) without contrast were normal. On the second day after admission, cerebral digital subtraction angiography (DSA) was performed, with results showing no abnormalities in the arterial phase, venous phase, or venous sinus phase.

Routine laboratory tests showed normal results except for elevated levels of serum creatinine (152.18 μmol/L), homocysteine (52.33 μmol/L), total cholesterol (5.71 mmol/L), low density lipoprotein cholesterol (3.77 mmol/L), and plasma D-Dimer (3.52 μg/ml). Serum tumor markers were negative. The results of hereditary thrombophilia screening revealed an obviously decreased activity of plasma protein S (41.2%). The patient’s son and daughter also underwent tests for plasma protein S activity, and both showed decreased activity (15.2 and 10.0%, respectively). Cardiac workup including color Doppler echocardiography and Holter monitoring revealed no abnormalities. Chest CT plain scans showed no lung abnormalities. The results of cerebrospinal fluid analysis were within normal limits, with no evidence of viral, bacterial, or fungal infections. Doppler ultrasonography revealed residual venous thrombosis in the right popliteal and posterior tibial veins. Affected leptomeningeal and cortical biopsies were further performed after an informed consent form was obtained from the patient. Hematoxylin and eosin staining revealed no inflammatory changes in the blood vessels. When stained with Congo red and viewed under a polarizing microscope, no amyloid deposition in the vessel walls was found.

Finally, the patient was diagnosed with MCVD, hypertension, hereditary protein S deficiency (PSD), hyperhomocysteinemia, and hypercholesterolemia. Following treatment with antihypertensive drugs, folic acid, vitamin B12 and statins, the patient’s blood pressure and levels of serum homocysteine and cholesterol were controlled to within the normal range. Their modified Rankin Scale (mRS) score was 2 at discharge. Warfarin was administered as a secondary prophylaxis for venous thromboembolism 2 months after the onset of stroke; however, the patient did not adhere to the monitoring scheme of blood coagulation function. Therefore, antiplatelet therapy with clopidogrel was given as an alternative, due to the patient’s refusal of other oral anticoagulants. Consequently, he occasionally had symptomatic recurrence of lower limb DVT during the 5-year follow-up period, yet with no recurrence of ICH nor CMBs visible on the follow-up head SWI. The degree of WMH was steady at Grade II on the Fazekas scale, and the mRS score was maintained at 2 during the follow-up, with no clinically apparent cognitive impairment. The patient also suffered hoarseness starting at the age of 66 years; a laryngoscope was performed at that time with no abnormalities found. About 1 year later, the patient had dry cough and received a chest CT exam due to an episode of hemoptysis, and a mass in the upper lobe of the left lung was discovered. He was subsequently diagnosed with lung cancer, and the patient died of the disease at 68 years of age.

## Discussion and conclusions

The patient had an acute hemiplegia followed by an episode of focal clonic seizure, with a CT scan showing two hyperdensities in the bilateral parietal lobes and a mixed density the left frontal lobe. The most likely nature of the lesions was deemed to be stroke with multiple parietal hemorrhages and a left frontal infarction with hemorrhagic transformation. A positive family history combined with a past history of lower limb DVT should first raise suspicion for cerebral venous thrombosis, which can present acutely with multi-type parenchymal brain lesions, including brain swelling, edema, ischemia, venous infarction or hemorrhage, or hemorrhagic venous infarction. Among the other etiologies of ICH, cerebral amyloid angiopathy (CAA) should also be considered due to the presence of multiple spontaneous lobar hemorrhages in elderly patients. A history of Raynaud’s phenomenon suggests a possibility of vasculitis. Moreover, vascular malformations and moyamoya disease as uncommon etiologies of ICH cannot be ruled out. ICH caused by brain tumors, cardiogenic cerebral embolism due to endocarditis, atrial fibrillation or patent foramen ovale, and coagulation disorders secondary to thrombosis or the use of anticoagulants or thrombolytics can be ruled out, due to the normal head CT prior to illness onset and the lack of supporting medical histories or results of auxiliary examinations.

However, the negative findings of brain MRV, SWI and DSA excluded the possibility of cerebral venous thrombosis, vascular malformations or moyamoya disease. Therefore, CAA and vasculitis were taken into consideration. Unexpectedly, pathological findings failed to support a diagnosis of CAA and vasculitis. A negative finding of CMBs was additionally inconsistent with the possibility of CAA, due to a correlation between SWI-identified CMBs and CAA-related pathologies [3].

Recurrent thrombotic events can easily bring to mind “thrombophilia”; thus, hereditary thrombophilia screenings were performed due to the family history of DVT. As both the patient and his children displayed markedly decreased plasma protein S activity, a diagnosis of hereditary PSD was warranted. Besides PSD, the patient had other risk factors for thrombophilia, namely hyperhomocysteinemia and occult lung cancer that had possibly been in existence at the onset of stroke, despite the normal lung CT plain scan results at that time. These factors, alone or in combination, can result in a hypercoagulable state, which is a recognized etiology for both venous and arterial thrombosis. Furthermore, both hypertension and hypercholesteremia are independent risk factor for arterial thrombosis. Among them, hypertension is also the most common attributable risk factor for primary ICH.

This patient had simultaneous multiple ICH (SMICH). SMICH is an infrequent form of ICH, occurring in less than 5.9% of all primary ICH cases [4–6]. As with solitary ICH, hypertension is still the most important etiological factor for SMICH [7]. A comprehensive review revealed that nearly all of the reported cases of primary SMICH were associated with hypertension [8]. Hypercholesterolemia, hyperhomocysteinemia, and systemic cancer have also been identified to be clinically associated with an increased risk of ICH [7, 9, 10]. Among them, hyperhomocysteinemia has been shown to be experimentally associated with endothelial link dysfunction in the autoregulation of cerebral blood flow [11]. Concerning the presence of Raynaud’s phenomenon in this case, it may be attributable to PSD due to the involvement of impaired fibrinolysis in the pathogenesis of Raynaud’s phenomenon, as it may predispose arteries to fibrin deposition and vascular obstruction [12].

We speculate that hypertension, PSD, hyperhomocysteinemia and hypercholesterolemia are the core causes of MCVD in this case. Hypertensive cerebral vascular lesions can induce both hemorrhagic stroke and ischemic stroke owing to hyalinosis and fibrinoid necrosis of the walls, as well as formations of microaneurysms or hyperplasia of the internal and external layers of the cerebral arterioles and small arteries. Furthermore, hyperhomocysteinemia and hypercholesterolemia serve as aggravating factors in atherosclerosis. PSD and the underlying occult lung cancer add further insult to injury by promoting hypercoagulability, as well as alterations of the immune system associated with an impairment of the coagulation system [13]. Immune system abnormalities could lead not only to recurrent cerebrovascular and systemic vascular events – including hemorrhagic and ischemic strokes and DVT [14] – but also, along with hypercoagulability, to the development of occult cancer [15], which may not be immediately detectable on regular diagnostic imaging. An initial stroke could result in another lesion of the same or opposite nature within a short time, this phenomenon could be explained by “biphasic hypothetical mechanism” [16].

In conclusion, this case presents complex underlying mechanisms of MCVD, in which hypertension and hyperhomocysteinemia are considered to play a central role.

## Abbreviations

CT: Computed tomography CMBs Cerebral microbleeds CAA Cerebral amyloid angiopathy DWI Diffusion-weighted imaging DVT Deep venous thrombosis DSA Digital subtraction angiography FLAIR Fluid attenuated inversion recovery ICH Intracerebral hemorrhages MCVD Mixed cerebrovascular disease MRI Magnetic resonance imaging MRA Magnetic resonance angiography MRV Magnetic resonance venography mRS Modified rankin scale PSD Protein s deficiency SMICH Simultaneous multiple intracerebral hemorrhages WMH White matter hyperintensity
